# Web Axillary Pain Syndrome—Literature Evidence and Novel Rehabilitative Suggestions: A Narrative Review

**DOI:** 10.3390/ijerph181910383

**Published:** 2021-10-02

**Authors:** Francesco Agostini, Carmine Attanasi, Andrea Bernetti, Massimiliano Mangone, Marco Paoloni, Edoardo del Monte, Massimo Mammucari, Enrica Maggiori, Domenico Russo, Raffaele Di Marzo, Alberto Migliore, Teresa Paolucci

**Affiliations:** 1 Department of Anatomical and Histological Sciences, Legal Medicine and Orthopedics, Sapienza University, 00185 Rome, Italy; andrea.bernetti@uniroma1.it (A.B.); massimiliano.mangone@uniroma1.it (M.M.); marco.paoloni@uniroma1.it (M.P.); 2 Physical Medicine and Rehabilitation Unit, Santa Caterina Novella Hospital, 73013 Lecce, Italy; carmine.attanasi@gmail.com; 3 San Camillo Forlanini School of Physiotherapy, 00152 Rome, Italy; delmonte.1740447@studenti.uniroma1.it; 4 Primary Care Unit ASL RM 1, 00193 Rome, Italy; massimo.mammucari@libero.it (M.M.); enricamaggiori@libero.it (E.M.); 5 San Marco Hospice and Palliative Care, 04100 Latina, Italy; dt.domenico.russo@tiscali.it; 6 Italian Society of Mesotherapy, 00185 Rome, Italy; dimarzoraffaele@gmail.com; 7 Unit of Rheumatology, San Pietro Fatebenefratelli Hospital, 00186 Rome, Italy; migliore.alberto60@gmail.com; 8 Department of Oral Medical Science and Biotechnology, G. D’Annunzio University of Chieti-Pescara, 66100 Chieti, Italy; teresa.paolucci@unich.it

**Keywords:** physiotherapy, axillary web syndrome, rehabilitation, breast cancer, lymphadenectomy, mesotherapy

## Abstract

Axillary web syndrome (AWS) is defined as a visible and palpable network of cords in the skin of the axillary cavity that are tensed by shoulder abduction following surgery for breast cancer, causing significant functional limits of the ipsilateral upper limb (UL) and pain. The purpose of this narrative review is to discuss rehabilitation approaches for greater efficacy with respect to pain and novel suggestions. AWS is a frequent complication of axillary lymphadenectomy that necessitates a thorough follow-up in the medium to long term. Physiotherapy is effective in the treatment of functional limb deficits, the management of pain, and the treatment of upper limb disability. The best management approach involves the use of soft tissue techniques to slow the natural course of the syndrome, in association with therapeutic exercises for functional recovery and muscle strengthening. AWS is linked secondary lymphedema, requiring integration with manual lymphatic drainage. The physiotherapy management of AWS is currently fragmented, and insufficient information is available on the nature of the disease. Thus, randomized and controlled studies that compare rehabilitation approaches in AWS are desirable, including the possibility of using mesotherapy in the treatment of axillary and upper limb pain.

## 1. Introduction

Axillary web syndrome (AWS) was defined in 2001 by Moskovitz et al. as a visible and palpable network of cords in the skin of the axillary cavity, tensed by shoulder abduction following surgery for breast cancer, significantly limiting the function of the ipsilateral upper limb (UL) and causing pain [1]. Based on this definition, several names have been derived for this syndrome, often referred to as cording, axillary string, vascular string, and lymphatic cord [2].

AWS is also called Mondor disease, attributed to superficial sclerosing venous thrombophlebitis [3,4,5]—the pathophysiology is angiolymphatic and fibrotic in origin, with lymphatic and fibroblastic involvement, and the cord can be exacerbated by tightness of the surrounding tissue [4,5,6,7]. The cording usually lies in the region of the axillary cavity and can extend along the medial surface of the upper arm and the ipsilateral lateral chest wall in 20% of cases [8], resulting in mechanical limitations in shoulder abduction; it is not uncommon that the cord extends to the forearm, occasionally reaching the radial tuberosity of the wrist [9,10]. 

Breast cancer (BC) survivors are more susceptible to AWS after breast reconstruction, with an incidence of 48.8% [11] versus 28.1% after surgery without reconstruction, and pain in the ipsilateral upper limb that is associated with AWS has been reported in 5.4% of patients; shoulder joint restriction is observed in 11.4% [12]. AWS occurs more frequently in the 2- and 8-weeks post-surgery phases, but its onset can be late, from 11 weeks to months following surgery, with a variable frequency and close relation to lymphadenectomy. 

Early diagnosis and early rehabilitation are essential to reduce upper limb pain, recover the range of shoulder motion, and dissipate the axillary cord [13]. Crane P. et al. reported good effects of thoracic manipulation and stretching as a rehabilitative treatment [14]. The literature suggests that manual lymphatic drainage (MLD) be combined with soft tissue mobilization and exercises for greater efficacy [15]. However, whether MLD reduces the risk of lymphedema is still debated, and a recent review by Mining Liang et al. [16] stresses the need for further studies in this regard.

Although AWS appears to be as frequent and detrimental as other morbidities after BC surgery and treatment, few studies have focused on this problem. Thus, it must be considered that AWS is often associated with other sequelae, including myofascial dysfunction, frozen shoulder, lymphostasis, postmastectomy syndrome, and donor site morbidity following breast reconstruction—rendering rehabilitative management critical [17]. There are no rehabilitative guidelines on which to base therapeutic interventions: current protocols differ in the proposed rehabilitation techniques, and clear references are missing with regard to the duration of the rehabilitation. Thus, regarding the importance of rehabilitation in the treatment of AWS, the purpose of this narrative review is to indicate rehabilitation approaches to increase efficacy with respect to pain.

## 2. Research Strategies

This narrative review was performed by a scientific literature research (Medline, PEDro, Database Cochrane and Scopus) from 1 June 2020 to 5 December 2020, using the following Mesh terms (Axillary Web Syndrome AND pain) OR (Axillary Web Syndrome AND upper limb function) OR (Axillary Web Syndrome AND breast cancer) OR Axillary Web Syndrome AND lymphedema) OR (Axillary Web Syndrome AND mesotherapy) OR (Axillary Web Syndrome AND exercise) OR (Axillary Web Syndrome AND Physiotherapy) OR (Axillary Web Syndrome AND manual therapy). The research included articles dealing with the rehabilitative management of patients suffering from Axillary Web Syndrome. Three researchers in physical and rehabilitation medicine conducted data extraction independently and the inconsistencies were overcome by comparison of the data and the debate. Articles with a mean age of the patients between 18 and 70 years were considered. Documents that have been excluded include those where it was not possible to find the complete text, those not in English, those with non-rehabilitative topics, and those with a methodology not adequately described or that did not provide evidence-based elements. The outcomes considered were pain, function, and disability. 

## 3. Rehabilitation: Functional Recovery and Pain Management

The first study on this topic was reported by Alexander H. Moskovitz in 750 patients who underwent breast surgery, showing that 6% of patients presented with “cording” that was consistent AWS, with a self-limiting trend and spontaneous resolution from 2 to 3 months [1]. According to the most recent findings, referring to the systematic review of S. Yeung et al. [2], there is still no clear evidence to define a single clinical presentation or the rehabilitative path in the management of AWS. 

Of the 37 studies in the review by S. Yeung, only two—by Moskovitz and Leideinus [1,18]—reported the absence of any efficacy of physical therapy in resolving symptoms; the remaining 35 studies contend that physiotherapy is important, despite differing in lengths of efficacy. Koehler et al. [8] opined that AWS persists and that its clinical presentation appears in 59% of patients after BC surgery after 12 weeks, in contrast with Moskovitz et al., who reported spontaneous resolution at 3 months after surgery. Dinas et al. [10] showed that rehabilitation was effective in recovering range of motion (ROM) of the shoulder and effecting cord resolution for up to 6–8 weeks, in accordance with Moskovitz and Leindeinus who underlined how shoulder mobilization and fascial mobilization exercises could accelerate the healing process and contain painful symptoms. Although there are no RCTs that compare physical therapy with placebo, physiotherapy is effective in reducing symptoms and recovering function, slowing AWS. 

## 4. Physical Therapy: What Is the Best Approach?

What is the most effective rehabilitative technique in managing the functional limitations of the upper arm and pain in AWS patients? There are no defined rehabilitative guidelines or rehabilitative protocols or RCTs that have shown the superiority of a rehabilitative technique in AWS over another. To date, Yeung et al. [2], reviewed eight studies and recommended the increasing administration of therapeutic exercises to patients for better care for AWS patients. 

### 4.1. Exercise

Depending on AWS severity, exercises should increase in intensity from load and gravity-assisted training to resistance exercises; similarly, muscle stretching should first be passive (executed by an expert physiotherapist in breast cancer rehabilitation), becoming active with stretching exercises for the pectoralis major and minor, biceps brachii, triceps brachii, dorsalis magnus, rotator cuff muscles, and cervical-dorsal region. Postural treatment, as suggested by the study, can include active exercises, postural recommendations for the trunk and shoulders, and diaphragmatic exercises. 

A revision by Koeheler et al. [8] highlighted the role of the physiotherapist with respect to the educational program, which comprises an initial illustration of the exercises with the therapist and independent replication at home. During the exercise program, articular mobilization can be integrated with exercise to reduce secondary restriction of the shoulder. In patients who understand and repeat the correct execution of the exercise, a home program could be amplified with auto-manipulation of the adherence and cord. Josenhans [19] demonstrated that patient self-observations of the cord and volume of the upper arm are a good practice to identify redness and swelling, to integrate subsequent treatment with lymphatic drainage techniques. 

### 4.2. Manipulative Treatment

Concerning tissue adherence of the cord, which is the main limitation of this disease, soft tissue techniques, such as myofascial manipulation, are recognized as the chief effective treatment to manage cordal tissue limitations. A manual approach, combined with therapeutic exercises [2,8,19], is an excellent rehabilitative alliance for the treatment of AWS for functional recovery and pain reduction. Several manual techniques are described in the literature, such as myofascial release, scar tissue massage, and lymphatic manual drainage. Myofascial techniques often include cord stretching and passive myofascial traction. 

Josenhans et al. [19] observed that manual myofascial techniques increase the range of motion in flexion and abduction of 20–40° of the shoulder for each session of treatment. After manual treatment, the “cord” tends to produce an audible sound that is painless and without collateral effects, followed by symptom relief and greater long-term articulation. This “sound” (reported by many authors as a “pop” or “snap”) is hypothesized to be produced by the rupture of the adherence after tension or by new connective tissue disruption on the lymphatic vessel surface. 

The breaking cord theory has not been confirmed by long-term follow-up studies. Many experts claim [8,9] that it is incorrect to discuss actual rupture of the cord, because the disruption occurs in the inflamed fibrotic tissue that envelops it, as a direct consequence of the surgery of axilla structures. The study by Yeung et al. [2] represents the only case report in which a patient experienced bruising after 24–48 h after manipulative session.

The etiopathogenesis of the cord has not been determined; thus, the effect of cord rupture is unknown. For this reason, a slow and progressive approach with painless and soft maneuvers is recommended [8,9,19]. 

A rare case in which manual therapy is strongly contraindicated is when the patient is undergoing radiotherapy or when they present with axillar metastases. Therapy must be interrupted for up to 2 weeks after the end of the radiotherapy cycle and spared from the region in which secondary neoplasia is diagnosed. The physiotherapist cannot cause pain by manipulating the axillary region, because a pain-free approach is strongly recommended; moreover, an aggressive approach can cause an inflammation [20]. 

Concerning manipulation treatment, Fourie and Robb [21] discussed the proper technique suggesting a lighter touch to start the treatment and subsequently, a deeper fascial mobilization of the tissues. A case report by Kepics [22] described a rehabilitation approach for AWS patients, emphasizing the importance of a cautious approach, especially in the early rehabilitation phases, which coincide with inflammation and acute pain, consistent with previous studies. Fascial manipulation is performed in a peripheral-proximal direction by positioning the patient’s limb in slight abduction with the elbow as extended as possible and the forearm supinated; after being positioned, the patient is asked to perform flexion and extension movements of the wrist. Depending on the extension of the cords, this active mobilization could be performed in various degrees of abduction. In the same position, myofascial techniques and transverse connective tissue massage are applied to the adherent areas of the cord and surgical scar. The first technique consists of the manipulation of connective tissue, lasting approximately two-thirds of the session, paying attention not to concentrate it on a single point for a prolonged time to avoid complications such as lymphedema and hyperemia. The cord is then mobilized and placed in “stretch” [19,20,22]. Sometimes, a therapist stabilizes the patient’s dorsal region while another performs traction in neutral rotation flexion of the upper limb: this technique is effective for those who have one or more cords with distal or thoracic extension. This approach is more comfortable and less painful for the subject than classic manipulations.

## 5. Timing of Rehabilitative Settings

From an analysis of the literature, there are no sufficient data to conclude what the optimal postoperative timing is to start rehabilitation. According to data by Josenhans [19], only 50% of patients undergo rehabilitation within 4 weeks of surgery, whereas the remaining 50% delay the start of therapy by several months to years after surgery (33% after 1–3 months and 17% from 1 to 11 years). The reasons for such a large gap in timing are unknown [23]. 

Based on the literature, rehabilitation for a diagnosis of AWS should last 4–5 weeks, with 2–3 sessions per week with an average duration of 30–40 min for each session. Particular attention is given to patients with severe restrictions to upper limb function and those who must undergo radiotherapy, who will be forced to interrupt therapy for the entire duration of the cycle; in these specific cases, patients should follow an intensive rehabilitation session (up to 5 sessions per week) [2,15,19,21,22,23]. 

A home program of gentle stretching and self-mobilization was taught and modified at each treatment session. Treatment goals were to increase and restore tissue mobility and reduce restrictions in soft tissue glide. Feedback, re-assessment of range of motion, and tissue glide were incorporated into all sessions [21], as well as gentle circular mobilization of the identified tissue tightness on the chest wall with full hand contact and touch, and longitudinal tissue stretch to strain the tight cords with the patient’s arm in available abduction [21].

In conclusion, given the high variability in the timing of the onset of AWS, it is not possible to determine the optimal postoperative day to begin rehabilitation; in contrast, if treated, AWS varies from 4 to 5 weeks in approximately 80% of patients, whereas the remaining 20% requires extension of the rehabilitation intervention beyond 3 months. According to the evidence, in those who do not take advantage of rehabilitation, the AWS persists for 3 to 18 months; however, there are no reports on the percentage of functional recovery in patients who do not undergo rehabilitation (see Table 1). 

## 6. Axillary Web Syndrome and Lymphedema

Breast cancer-related lymphedema (BCRL) is one of the most frequent secondary complications in breast-operated patients, particularly in patients who undergo ALND axillary surgery and radiotherapy [24,25,26,27]. Like AWS, BCRL is associated with the lesion of the lymph nodes and related vessels that affect the ipsilateral upper quadrant. Roughly thirty percent of BC survivors will have AWS during their first year of survival. As institutions prioritize screening efforts, early postoperative prospective surveillance is needed for women aged over 60 years due to a high risk for AWS and any woman with AWS for increased risk of lymphedema, based on our findings [24,27]. 

Given the lack of data on the pathophysiology of AWS-related lymphedema, Ryans et al. [24] examined the association between BCRL and AWS in a cohort of 354 patients and determined whether AWS is a risk factor for BCRL in the 3 years following surgery—the period within which lymphedema most frequently occurs. Of the 113 women with AWS, 46 (40.7%) had clinically documented lymphedema. However, this percentage is much higher than that reported by O’Toole et al. [3], recording an incidence of 16.2% at a median of 7.2 months after surgery, concluding that the axillary cord is an independent risk factor for limb volumetric augmentation, with a variation of ≥5% [24]. In fact, according to Ryans et al. [24], AWS patients have a 44% greater chance of developing lymphedema during the first postoperative year, whereas BC survivors who developed AWS in the first postoperative month were nearly three times more likely to develop lymphedema than other women with AWS. 

In a prospective study by Moskovitz et al. [1], of 750 patients who were undergoing ALND, 11% of subjects with AWS developed BCRL, also indicating that there is no correlation between axillary syndrome and secondary lymphedema. Consistent with Moskovitz et al., a follow-up report by Koheler et al. [24] concluded that AWS can persist for 18 months and longer, developing beyond the early postoperative period, and reoccur after resolution; thus, clinicians must be aware of the chronicity of AWS and its association with reduced range of motion and function. A prospective 10-year cohort study by Wariss et al. came to similar conclusions: in the follow-up of 964 patients, an incidence rate of 35.9% for AWS and 31.4% for BCRL emerged. Although these results were similar to those of Ryans et al., the authors of the follow-up concluded that there was no association between AWS and lymphedema in the 10 years following surgery [28]. 

Cho et al. [15] compared an AWS study group that was undergoing physical therapy (PT) and manual lymphatic drainage (MLD) with a control group that performed PT only. Their results suggested that pain and limb volume were significantly lower in the study group (physiotherapy and manual lymphatic drainage) versus the PT-only group. Despite O’Toole et al. [3] demonstrating that axillary cords are independent of the development of lymphedema, AWS is a syndrome that reflects lymphatic stagnation and thus benefits from manual drainage treatment, which must be integrated into a rehabilitation program that aims to increase limb function through progressive therapeutic exercises. Torres Lacomba et al. [29] support the use of MLD for preventing lymphedema in the two years following surgery for BC. Although AWS is a risk factor for lymphedema after BC surgery, a high-evidence study has examined the correlation between the two complications [15,28,30].

## 7. Novel Rehabilitative Suggestions: Mesotherapy Approach

Intradermal therapy (IDT), known as mesotherapy, injects a drug into the surface layer of the skin. In particular, it involves the use of a short needle to deposit the drug in the dermis. The intradermal microdeposit modulates the drug’s kinetics, slowing its absorption and prolonging the local mechanism of action. It has been applied successfully in the treatment of certain forms of localized pain syndromes and other local clinical conditions [31]. 

**Figure 1:**
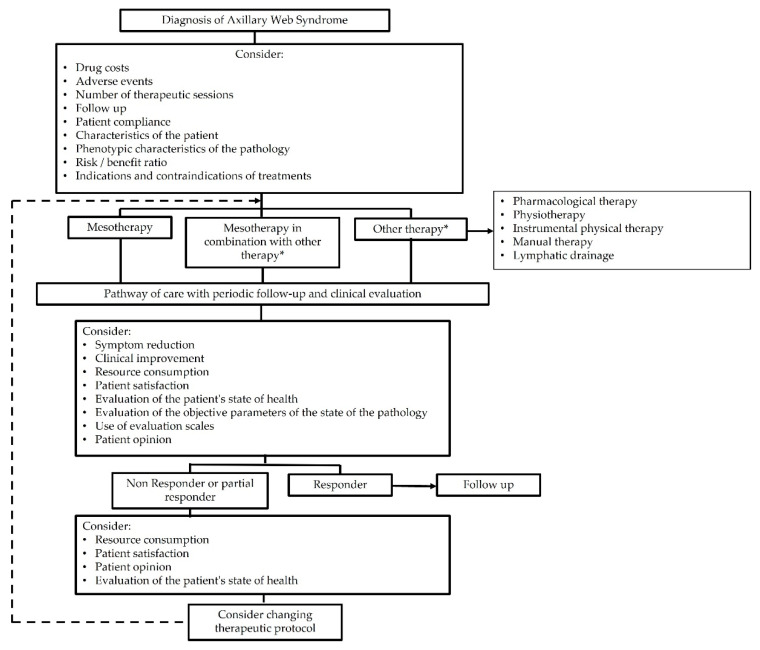
Algorithm for the treatment of pain in mesotherapy (intradermal therapy—IDT).

Mesotherapy, performed correctly according to aseptic procedures, is a valid resource in the treatment of pain in AWS, based in part on its dose-sparing effect. Mesotherapy is being used in the treatment of mild to moderate forms of lymphedema, with good results [31]. According to the recent recommendations of the Italian Society of Mesotherapy (SIM), IDT is beneficial immediately before physiotherapy techniques to facilitate joint mobility and reduce pain, and IDT should be considered in the management of localized pain syndromes in accordance with the best therapeutic path for each patient [32]. SIM proposes exploring the best treatment algorithm for AWS—rehabilitation alone versus rehabilitation + IDT, with functional recovery and pain management as endpoints (see Figure 1).

## 8. Conclusions

As a narrative review, the study presents a lack of “systematic” quantitative and qualitative analysis of the studies analyzed. However, it allowed us to synthesize and focus attention on the different rehabilitation approaches discussed in the literature on AWS and useful support to the clinician for a correct management of the BC patient and their sequelae. AWS is clinically identified as a frequent complication of axillary lymphadenectomy, necessitating a thorough follow-up in the medium to long term. From the articles included in this narrative review, physiotherapy appears to have a good effect in the reduction of pain and the recovery of limb function on the operated side on AWS.

The best management approach involves soft tissue techniques for reducing the natural course of the syndrome, in association with therapeutic exercises for functional recovery and muscle strengthening. If AWS is associated with secondary lymphedema, it will require the integration of manual lymphatic drainage. 

However, the rehabilitative management of AWS currently appears fragmented and there are no standardized treatment protocols. Therefore, future randomized and controlled studies should compare the different rehabilitation approaches in AWS and integrated therapies such as mesotherapy need to better discuss. 

## Figures and Tables

**Table 1:** Timing of rehabilitation and sessions.

Authors	Duration of the Session	Weekly Frequency	Duration of Rehabilitation Treatment
Yeung et al. [2]	30–45 min	2–3 sessions	From 2 to 5 weeks
Koheler et al. [24]	-	2–4 sessions	-
Cho et al. [15]	40 min (physical therapy) + manual lymphatic drainage; 70 min (physical therapy + manual lymphatic drainage)	3 sessions for physical therapy alone; 5 sessions for physical therapy + manual lymphatic drainage	4 weeks
Fourie and Robb [21]	30–45 min	-	3–4 weeks
Josenhans [19]	30 min	2–3 sessions	3–4 weeks
Lattanzi et al. [23]	-	From 3 sessions to 1 session at the end of treatment	5 weeks
Kepics et al. [22]	-	2–3 sessions	4 weeks

## Data Availability

All data are available in the manuscript.
